# Insurance Companies Return to the Five Dollar Fee

**Published:** 1907-09

**Authors:** 


### INSURANCE COMPANIES RETURN TO THE FIVE DOLLAR FEE.

---

We note with pleasure the return of some of the insurance companies. (The Equitable and Mutual Life of New York) to the former flat fee of five dollars for each examination. This the companies say was made practicable after six months trial of the lower rate by a re-arrangement of the first year's expense as provided by the Armstrong Insurance Laws of New York.

They admit there was much disorganization of the Medical Departments from the almost unanimous refusal of the Medical Examiners throughout the country to enter into the cut-rate contracts.. Many of their most eminent experienced field medical representatives resigned and they realized the danger of the risks they were assuming in allowing this condition to continue. It is expected that the other companies will soon make similar announcements.

The lesson to be drawn from this notable campaign for our rights is that in unanimity of action there is strength and that other much needed reforms might be procured if the entire profession took a determined stand and demanded them.

---

The city law affecting the sale of undrawn poultry has gone into effect and hereafter it is claimed that most of the poultry will be dressed here in Atlanta as it will be more profitable than to have them shipped into the city drawn.

